# Identification and Expression Profiling of Nonphosphorus Glycerolipid Synthase Genes in Response to Abiotic Stresses in *Dendrobium catenatum*

**DOI:** 10.3390/plants10061204

**Published:** 2021-06-13

**Authors:** Xinqiao Zhan, Yichun Qian, Bizeng Mao

**Affiliations:** 1 Institute of Biopharmaceuticals, Taizhou University, Taizhou 318000, China; 2 Institute of Biotechnology, Zhejiang University, Hangzhou 310000, China; ycqian1999@163.com

**Keywords:** nonphosphorus glycerolipid synthase, abiotic stress, circadian regulation, *Dendrobium*

## Abstract

*Dendrobium* *catenatum*, a valuable Chinese herb, frequently experiences abiotic stresses, such as cold and drought, under natural conditions. Nonphosphorus glycerolipid synthase (NGLS) genes are closely linked to the homeostasis of membrane lipids under abiotic stress in plants. However, there is limited information on *NGLS* genes in *D*. *catenatum*. In this study, a total of eight *DcaNGLS* genes were identified from the *D*. *catenatum* genome; these included three *monogalactosyldiacylglycerol synthase* (*DcaMGD1*, *2*, *3*) genes, two *digalactosyldiacylglycerol synthase* (*DcaDGD1*, *2*) genes, and three *sulfoquinovosyldiacylglycerol synthase* (*DcaSQD1*, *2.1*, *2.2*) genes. The gene structures and conserved motifs in the *DcaNGLSs* showed a high conservation during their evolution. Gene expression profiling showed that the *DcaNGLSs* were highly expressed in specific tissues and during rapid growth stages. Furthermore, most *DcaNGLSs* were strongly induced by freezing and post-freezing recovery. *DcaMGD1* and *DcaSQDs* were greatly induced by salt stress in leaves, while *DcaDGDs* were primarily induced by salt stress in roots. Under drought stress, most *DcaNGLSs* were regulated by circadian rhythms, and *DcaSQD2* was closely associated with drought recovery. Transcriptome analysis also revealed that MYB might be regulated by circadian rhythm and co-expressed with *DcaNGLSs* under drought stress. These results provide insight for the further functional investigation of NGLS and the regulation of nonphosphorus glycerolipid biosynthesis in *Dendrobium*.

## 1. Introduction

Plant cell membranes have specific, nonrandom glycerolipid compositions [1]. Chloroplasts mainly contain four glycerolipids, including high levels of the galactolipids monogalactosyldiacylglycerol (MGDG) and digalactosyldiacylglycerol (DGDG) and low levels of sulfoquinovosyldiacylglycerol (SQDG) and phosphatidylglycerol (PG) [2]. The thylakoids represent the largest membrane system in leaf mesophyll cells and contain approximately 52% MGDG, 26% DGDG, 6.5% SQDG, and 9.5% PG [2]. In plants, phosphate (Pi) deprivation has been reported to decrease the phospholipid content. Thus, nonphosphorus glycerolipids (NGLs) MGDG, DGDG, and SQDG play important roles in membrane lipid remodeling during the replacement of phospholipids [3]. Under Pi starvation, phospholipids are degraded and NGLs biosynthesis is usually activated in chloroplast membranes, but DGDG biosynthesis is also found in extraplastidic membranes and depends on DGDG synthase (DGD1) [4,5]. The disruption of *DGD1* results in dwarfed growth and reduced photosynthetic activity with an altered chloroplast ultrastructure because *DGD1* is responsible for 90% of DGDG biosynthesis [4]. The *dgd2* mutant experiences no further consequences for DGDG content or photosynthesis under normal conditions [6]. As with DGDG biosynthesis, *MGD1* is involved in the bulk of MGDG biosynthesis in chloroplasts, while *MGD2* and *MGD3* are strongly induced by Pi starvation and are responsible for an alternative galactolipid pathway in nongreen tissues such as roots and flowers [7]. In addition, *MGD1* is upregulated by light and cytokinins, while *MGD2* and *MGD3* are suppressed by cytokinins and induced through auxin signaling pathways [7,8]. These data describe the regulatory mechanisms of galactolipids that are involved in phytohormone signaling pathways and Pi-limitation responses.

SQDG is a unique anionic glycolipid in photosynthetic membranes that was first found to be involved in Pi starvation [5]. UDP-sulfoquinovose synthase (SQD1) uses sulfite and UDP-glucose to produce UDP-sulfoquinovose, and sulfoquinovosyltransferase (SQD2) transfers sulfoquinovose to diacylglycerol to generate SQDG [9,10]. SQD2 is also responsible for the synthesis of glucuronosyldiacylglycerol (GlcADG) in Arabidopsis [3]. Recently, one *SQD1* and three putative genes for *SQD2* were reported in rice [11]. In addition to SQDG synthase activity, *SQD2.1* overexpression can enhance plant tolerance to salinity and drought stress [11]. In addition, another *SQD2.2* showed detectable activity for SQDG synthesis but exhibited flavonoid glycosylation activity [12]. Although there are few reports about NGLs being involved in salinity and drought stress, some studies have revealed that NGL hydrolysis represents a unique response to freezing stress. More drastic lipid compositional changes occur during post-freezing phases, which involve a decrease in the plastidic galactolipids MGDG and DGDG [13]. SENSITIVE TO FREEZING 2 (SFR2) progressively transfers galactosyl residues from MGDG to different galactolipid acceptors, finally forming the nonpolar lipid triacylglycerol to accommodate organelle shrinkage after freezing [14].

*Dendrobium catenatum* (also known as *D*. *officinale*), which belongs to the family Orchidaceae, is a perennial herb. *D*. *catenatum* contains many medicinal components, such as polysaccharides, alkaloids, and flavonoids [15]. In the last 20 years, *D*. *catenatum* has been successfully cultivated and become an important economic crop in China [16]. As an epiphytic plant, the growth of *D*. *catenatum* usually requires a warm and humid environment. However, drought, cold, salinity, and other environmental stresses greatly restrict its growth, resulting in serious yield loss. Studies on the functional NGL response to abiotic stress are rare. Therefore, we screened and identified the candidate genes of NGLs and analyzed NGL gene responses to environmental stresses. These results provide insight into the molecular breeding of resistance into *D*. *catenatum*.

## 2. Results

### 2.1. Identification of DcaNGLSs in the D. catenatum Genome

**Figure 1:**
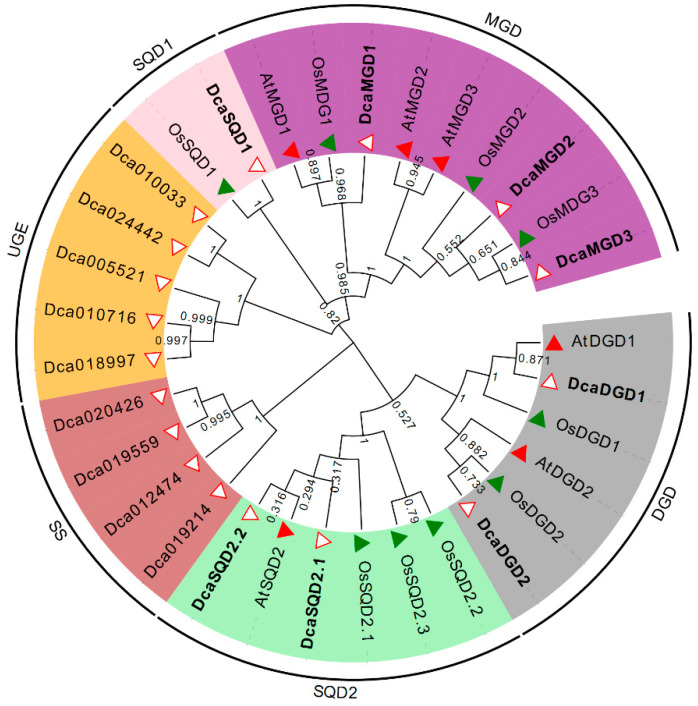
Phylogenetic analysis of NGLSs in *D*. *catenatum*, rice, and Arabidopsis. A total of eight NGLSs domain-containing proteins from *D*. *catenatum*, nine from rice, and six from Arabidopsis were used to construct the unrooted neighbor-joining (NJ) tree with a bootstrap of 1000 replicates. Four sucrose synthases (SS) and five UDP-glucose epimerases (UGE) were divided from the SQD1 and SQD2 clusters.

According to the NGLS homologs in the model plants Arabidopsis and *Oryza sativa*, a total of 18 candidate DcaNGLS proteins were originally obtained using a BLASTP search. Phylogenetic analysis showed the candidate proteins were classified into five clusters (Figure 1). Four sucrose synthases (SS) and five UDP-glucose epimerases (UGE) were identified from the SQD1 and SQD2 clusters. Finally, eight DcaNGLSs were confirmed and named after their Arabidopsis homologs. The basic information about the DcaNGLSs is shown in Table 1. The DcaNGLS proteins contain 303–794 AAs, with molecular weights of 34.6–90.6 kDa and isoelectric points of 5.91–9.01.

### 2.2. Gene Structures and Conserved Motifs of DcaNGLSs

**Figure 2:**
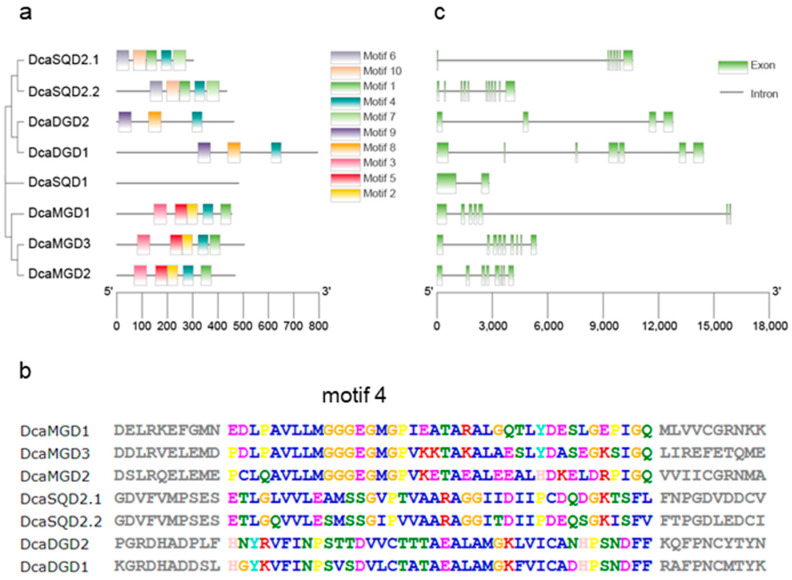
Domain organization and gene structure of DcaNGLSs. (**a**,**b**) The conserved motifs of DcaNGLSs are predicted by MEME. (**c**) The *DcaNGLSs* structures are constructed by GSDS 2.0.

To further understand the structure of DcaNGLS proteins, we searched for 10 conserved motifs in the DcaNGLSs with the MEME software. DcaMGD, DcaDGD, and DcaSQD2 displayed the same motif construct, and all contained motif 4 (Figure 2a,b). The exon-intron structures of *DcaNGLSs* shared a similar number of exons in the same clades (Figure 2c). Four long genes (>10 kb), *DcaMGD1*, *DcaDGD1*, *DcaDGD2*, and *DcaSQD2*.1, had much longer introns than their homologs, which could be related to the extension of the introns. 

### 2.3. Cis-Elements in the Promoter Regions of DcaNGLSs

**Figure 3:**
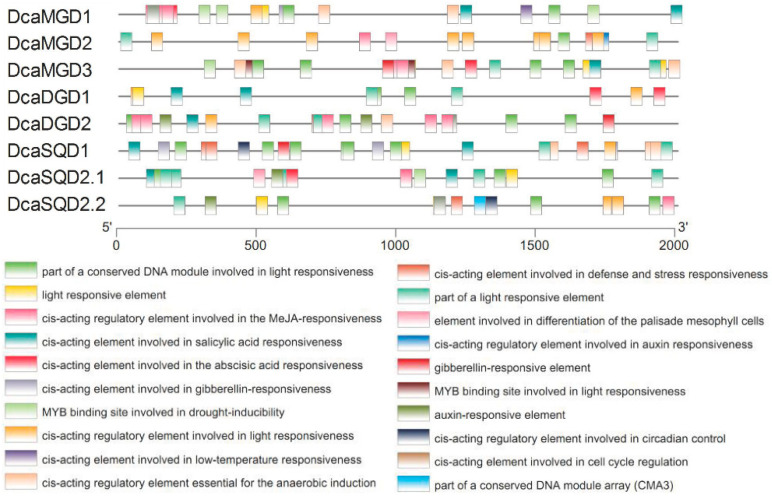
Predicted cis-elements in *DcaNGLS* promoters. Promoter sequences (−2000 bp) of 8 *DcaNGLSs* are analyzed by PlantCARE. The upstream length to the translation start site can be inferred according to the scale at the bottom.

Studies have shown that NGLS genes have diverse biological functions in plants, particularly in defense against drought stress, salt stress, and phosphate starvation [4,11,17]. To predict the putative functions of *DcaNGLSs* in response to biotic and abiotic stresses, we analyzed 2 kb upstream of the *DcaNGLSs* (Figure 3). Potential cis-elements in the promoter regions of *DcaNGLSs* were identified by the PlantCARE website. Twenty cis-elements were found in the promoter sequence of *DcaNGLSs*, such as abiotic stress-responsive cis-elements (e.g., drought inducibility and low temperature response) and phytohormone-related cis-elements (e.g., gibberellin, auxin, salicylic acid, abscisic acid, and MeJA). *DcaSQD1* and *DcaSQS2.2* contained circadian regulation-related cis-elements, and most *DcaNGLSs* contained light-responsive elements. These results suggest that *DcaNGLSs* may play important roles in abiotic stress responses and photoperiod regulation.

### 2.4. Tissue and Organ Expression Profiles of DcaNGLSs

**Figure 4:**
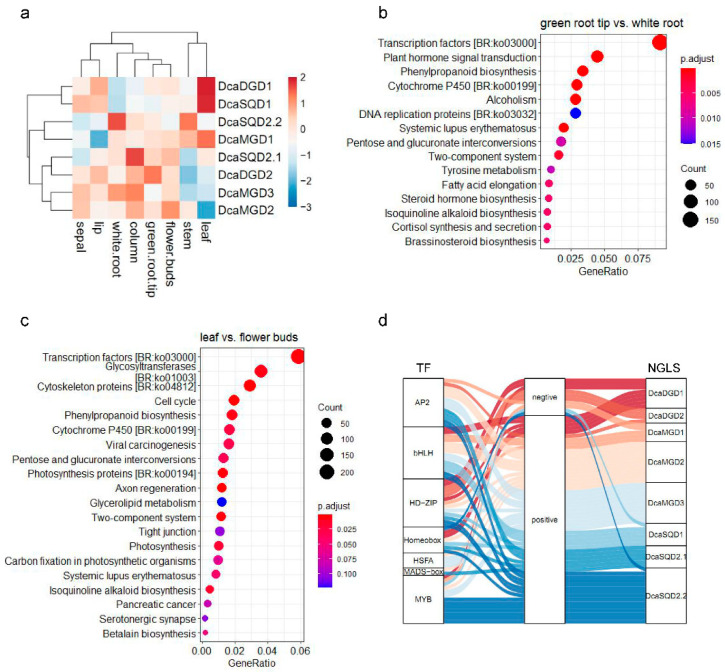
Expression patterns of *DcaNGLSs* in different tissues. (**a**) *DcaNGLSs* are highly expressed in the specific tissues. (**b**) KEGG enrichment analysis in green root tip vs. white root comparison. (**c**) KEGG enrichment analysis in leaf vs. flower buds comparison. (**d**) The Sankey diagram shows the regulatory relationships between transcription factors and *DcaNGLSs* leaf vs. flower buds. The color scale represents the color code for the log2 fold-change of gene expression in a.

To investigate the expression profiles of *DcaNGLSs* during growth and development in *D*. *catenatum*, RNA-seq data from different plant organs were detected [18]. Hierarchical clustering showed that most of the *DcaNGLSs* were specifically expressed in different tissues (Figure 4a). *DcaSQD1* displayed high expression levels in leaves, while *DcaSQD2.1* and *DcaSQD2.2* displayed high expression levels in columns and white roots, respectively. *DcaMGD1* and *DcaDGD1* displayed high expression levels in leaves, while *DcaDGD2*, *DcaMGD2*, and *DcaMGD3* were mainly expressed in nongreen tissues, such as roots and flowers. Secondary metabolism-related and phytohormone-related pathways were significantly enriched in green root tips vs. white roots and leaf vs. flower buds (Figure 4b,c). We further analyzed the correlation of transcription factors and *DcaNGLSs* in leaves vs. flower buds, finding that most transcription factors were positively related to the expression of *DcaNGLSs* (Figure 4d).

**Figure 5:**
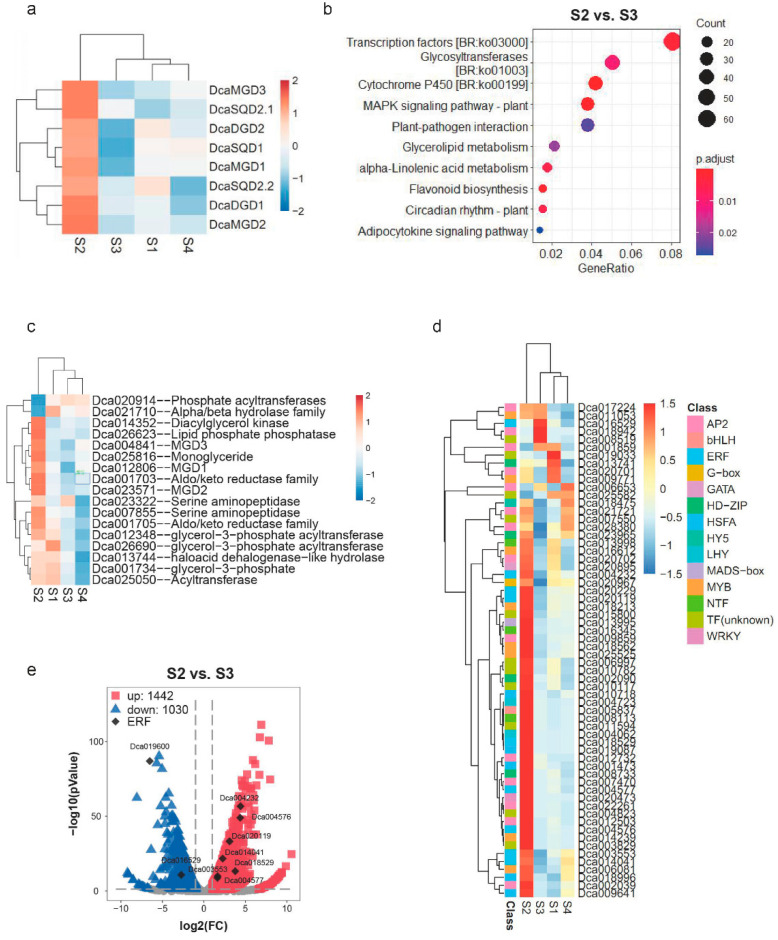
Expression patterns of *DcaNGLSs* in four stages. (**a**) *DcaNGLSs* are highly expressed in the S2. (**b**) KEGG enrichment analysis in S2 vs. S3 comparison. (**c**) Expression patterns of glycerolipid metabolism-related genes. (**d**) The expression levels of transcription factors in S2 vs. S3 comparison. (**e**). The expression distribution of ERF in S2 vs. S3 comparison. S1: plant experiences vegetative growth with few polysaccharides (4 months after sprouting); S2: plant accumulates polysaccharides rapidly (10 months after sprouting); S3: plant develops into a mature stage with the highest polysaccharide content (12 months after sprouting); S4: plant begins to die and the polysaccharide content decreases rapidly (16 months after sprouting). The color scale represents the color code for the log2 fold-change in gene expression in (**a**,**c**,**d**).

The expression patterns of *DcaNGLSs* were analyzed during four developmental periods of *D*. *catenatum* [19]. All *DcaNGLSs* displayed high expression levels in the rapid polysaccharide accumulation stage (S2, 10 months after sprouting) (Figure 5a). Furthermore, eight pathways were significantly enriched (*p* < 0.05) in the S2 vs. S3 (12 months after sprouting) comparison, such as “transcription factors” (*p* = 2.35 × 10^−3^), “flavonoid biosynthesis” (*p* = 9.00 × 10^−4^), and “cytochrome P450” (*p* = 3.85 × 10^−8^) (Figure 5b). Among them, seventeen glycerolipid metabolism pathway-related genes were investigated (Figure 5c), and most of the genes showed the highest expression in S2. Similarly, most transcription factors were predominantly expressed in S2, including AP2, bHLH, ERF, and MYB (Figure 5d). ERF is part of the large AP2/ERF multigene family and mediates physiological, developmental, and stress responses in plants [20,21]. Six ERF genes were significantly upregulated and two ERF genes were downregulated in S2 vs. S3 (Figure 5e). These results suggest that *DcaNGLSs* are associated with rapid polysaccharide accumulation regulated by phytohormone-dependent pathways.

### 2.5. Expression Levels of DcaNGLSs in Response to Abiotic Stresses

**Figure 6:**
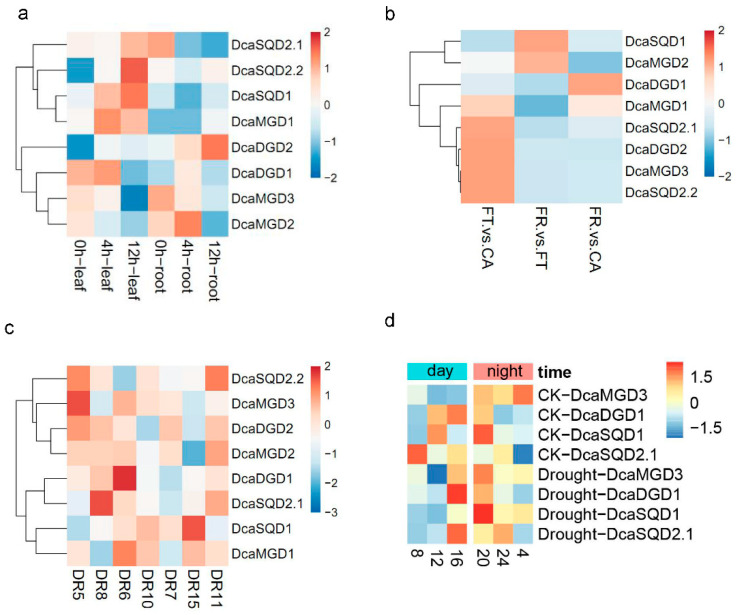
Expression of *DcaNGLSs* in response to abiotic stresses. (**a**) Expression of *DcaNGLSs* in response to salinity treatment. The seedlings were supplied with 250 mM NaCl, and the leaf and root sample were harvested at 0, 4, and 12 h. (**b**) Expression of *DcaNGLSs* in response to cold treatment. The 2-year-old plants were treated with cold acclimation (CA), freezing (FT), and post-freezing recovery (FR). (**c**) Expression of *DcaNGLSs* in response to drought. The seedlings were watered on the 1st day, dried from the 2nd to the 7th day, and re-watered on the 8th day. Leaves were collected at different times; DR5/DR8, DR6/DR10, and DR7/DR15 indicate sampling at 06:30 and 18:30 on the 2nd, 7th, and 9th days, respectively, and DR11 indicates sampling at 18:30 on the 8th day. (**d**). Effect of the time of day on *DcaNGLSs* expression induced by drought stress. The experiment was repeated three times with similar results. Color scale represents the color code for the log2 fold-change in gene expression. CK, control group.

**Figure 7:**
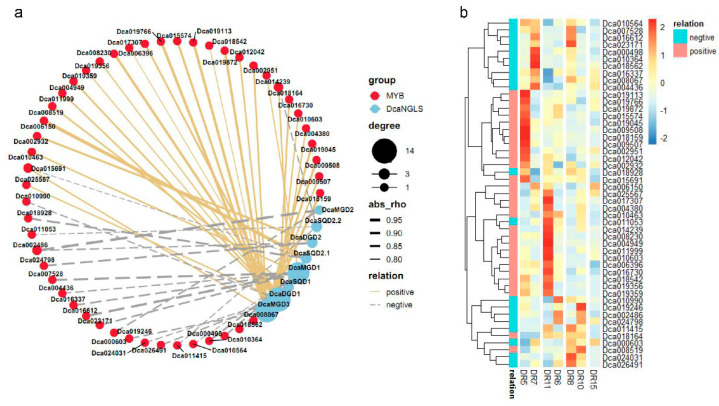
Co-expression analysis *DcaNGLSs* and MYB in response to drought. (**a**) Co-expression analysis of *DcaNGLSs* and MYB under drought stress. (**b**) The expression levels of MYB under drought stress. The color scale represents the color code for the log2 fold-change in gene expression.

Cis-element analyses revealed the putative functions of *DcaNGLSs* in response to abiotic stresses. We further studied the RNA-Seq data for the *DcaNGLSs* in response to salt, cold, and drought stresses (data sources described in methods). Hierarchical clustering showed that *DcaMGD1*, *DcaSQD1*, *DcaSQD2.1*, and *DcaSQD2.2* were primarily induced in leaves and that *DcaDGD2* was primarily induced in roots under salt stress (Figure 6a). Under cold stress, *DcaMGD1*, *DcaMGD3*, *DcaDGD2*, *DcaSQD2.1*, and *DcaSQD2.2* were upregulated in FT (freezing) vs. CA (cold acclimation), while *DcaSQD1* and *DcaMGD2* were upregulated in FR (post-freezing recovery) vs. FT (Figure 6b). For drought stress, the expression of most *DcaNGLSs* was not always the same under drought stress at dawn and dusk (Figure 6C). For instance, *DcaDGD2*, *DcaMGD3*, and *DcaSQD2.2* were induced on DR5 (sampling at 06:30 on the 2nd days), while *DcaDGD1* and *DcaSQD2.1* were induced on DR6 (sampling at 06:30 on the 7th days) and DR8 (sampling at 18:30 on the 2nd days), respectively. In addition, rewatering restored the expression levels of *DcaSQD2.1, DcaSQD2.2*, and *DcaDGD1*. Furthermore, we conducted a RT-PCR validation of the expression of four genes (*DcaMGD3*, *DcaDGD1*, *DcaSQD1*, and *DcaSQD2.1*) under drought conditions (Figure 6d). Four *DcaNGLSs* displayed obvious differences in expression at different times of day under control conditions. *DcaMGD3* was significantly elevated at night, while *DcaDGD1*, *DcaSQD1*, and *DcaSQD2.1* were active during the day. Interestingly, four *DcaNGLSs* displayed similar expression patterns and peaked at 16:00 and 20:00 under drought stress. Moreover, MYB genes played roles in response to abiotic stresses [22]. Co-expression analysis showed that 29 pairs had a positive correlation and 23 pairs were negatively correlated (Figure 7a). Two clusters of MYB genes were significantly upregulated in DR5 and DR11 (sampling at 18:30 on the 8th day) (Figure 7b). These results indicated MYB might be regulated by circadian rhythm and co-expressed with *DcaNGLSs* under drought stress.

## 3. Discussion

In higher plants, the thylakoid membrane is the site of the photochemical and electron transport reactions of oxygenic photosynthesis [23]. The biogenesis of thylakoid membranes is closely linked to the development of chloroplasts from other plastids such as proplastids. The thylakoid membranes are composed mainly of four glycerolipids, MGDG, DGDG, SQDG, and PG [2]. The disruption of thylakoid membrane lipids is fatal to photosynthetic growth. For instance, the final step in MGDG biosynthesis occurs in the plastid envelope, and *mgd1* lacks galactolipids and disrupts chloroplast structures, leading to the complete impairment of photosynthetic growth and embryogenesis [24]. In contrast to phospholipids, MDGD, DGDG, and SQDG are special NGLs that make up approximately 83% of chloroplast lipids [2]. However, unlike other families, the NGLS family does not have a strictly conserved domain. Except for SQD1, NGLSs all belong to the glycosyltransferase family, as shown in the CAZy database (http://www.cazy.org, (accessed on 31 May 2021)), and contain motif 4 (Figure 2). MGDG synthases are members of glycosyltransferase family 28 (GT28) and produce β-anomeric linkages. DGDG synthases and SQDG synthases belong to family GT4 and produce α-glycosidic linkages [2]. In Arabidopsis, MGDG synthases are classified into two types, A (MGD1) and B (MGD2 and MGD3) [7]. In our results, MGD1 in Arabidopsis, rice, and *D*. *catenatum* was grouped into one cluster. MGD2 and MGD3 in Arabidopsis were separated in rice and *D*. *catenatum* (Figure 1). This suggests that the relationship between rice and *D*. *catenatum* is closer than their relationship with Arabidopsis; the former two are both monocotyledons. 

Unlike other NGLSs, SQD1 contains an epimerase domain and produces sole glycosyl donors for SQDG synthesis [9]. Previous studies showed that *SQD1* expression increased under Pi starvation [25], but the loss of *SQD1* had no effect on GlcADG biosynthesis and growth phenotypes under Pi starvation [3]. In rice, the disruption of *SQD1* also had no effect on growth under salt stress [11]. However, the replacement of phospholipids with SQDG is a typical adaptive mechanism under Pi starvation [5]. Transcriptome analysis showed that *DcaSQD1* expression increased under salt and drought stresses and was upregulated in FR vs. FT (Figure 5). These results suggest that, as a limiting reaction in SQDG biosynthesis, *SQD1* expression may be necessary to maintain the balance of anionic thylakoid lipids under abiotic stresses.

Our research found that most *DcaNGLS* promoters contain MYB-binding cis-elements (Figure 3). MYB regulators participate in multiple biological processes and mediate phenylpropanoid metabolism under abiotic stress [26,27]. MYB is also a tool for metabolic engineering to control the transcriptional regulation of anthocyanin structural genes [28]. Transcriptome analysis showed that the expression of most *DcaNGLSs* was positively correlated with MYB expression in leaf vs. flower buds (Figure 4d). Similar to other orchids, *D. catenatum* displays unique flower morphologies and a variety of flower colors [29]. Under drought stress, the expression of 29 MYB genes was positively correlated with *DcaNGLS* expression and regulated by circadian rhythms (Figure 7). In addition, most MYB and *DcaNGLSs* displayed high expression levels in S2 (Figure 5a,d). *D*. *catenatum* accumulated polysaccharides rapidly in S2 [19]. These results suggest that MYB regulates *DcaNGLSs* during flower development, carbohydrate metabolism, and drought responses.

Although many studies have focused mainly on the regulation of NGLSs under Pi starvation and during photosynthetic activity, NGLSs are also related to salinity and drought stresses [11]. Similar to phosphate starvation, a lack of water enhanced MGDG and DGDG biosynthesis [30]. As drought stress continued, the two predominant molecular species, MGDG (36:6) and DGDG (36:6), began to decline [31]. Rice SQD2.1 mediates the glycosylation of flavonoids that are required for osmotic stress tolerance [11]. In our results, *DcaNGLSs* were strongly induced by salinity, cold, and drought stresses (Figure 6). Specifically, the expression levels of *DcaNGLSs* were significantly regulated by circadian rhythms under drought stress (Figure 6c). Our results also showed that the expression levels of *DcaMGD3*, *DcaDGD1*, *DcaSQD1*, and *DcaSQD2.1* all peaked at 16:00 and 20:00 under drought stress (Figure 6d). These results suggest that *DcaNGLSs* are involved in responses to abiotic stresses and are strongly influenced by the biological clock under drought stress.

## 4. Materials and Methods 

### 4.1. Identification of DcaNGLS Family in D. catenatum, Arabidopsis, and Oryza Sativa

The NGLS sequences, including genomic DNAs, CDS, and proteins in *Oryza sativa*, and Arabidopsis were retrieved from Phytozome v12 (https://phytozome.jgi.doe.gov/pz/portal.html, (accessed on 1 May 2021)). The genome of DcaNGLS was downloaded from NCBI (https://www.ncbi.nlm.nih.gov/genome, (accessed on 1 May 2021)). The DcaNGLS proteins were originally obtained using BLASTP search in NCBI. All of the hits were further confirmed in SMART (http://smart.embl-heidelberg.de/, (accessed on 1 May 2021)). 

### 4.2. Analysis of Phylogenetic Relationship, Motif Architecture, Gene Structure, and cis-Elements of Promoters

The sequences of NGLS proteins from these species were aligned with ClustalW software, and then an un-rooted tree was obtained with MEGA 7.0 software using a neighbor-joining method with a bootstrap of 1000 replicates [32,33]. The motif analysis was performed in MEME (https://meme-suite.org/meme/, (accessed on 12 January 2021)). The intron-exon distribution pattern of NGLS genes were reconstructed by GSDS 2.0 (http://gsds.cbi.pku.edu.cn/, (accessed on 12 January 2021)). The cis-elements of *DcaNGLS* promoters were analysed in PlantCARE (http://bioinformatics.psb.ugent.be/webtools/plantcare/html/, (accessed on 12 January 2021)). 

### 4.3. In Silico Expression Profiling of DcaNGLSs

For the tissues and stages expression profiling of *DcaNGLSs*, the raw RNA-seq data (PRJNA348403 and PRJNA277909) were downloaded from the NCBI [18,19]. The raw RNA-seq data of drought stress were described and obtained from Sci Data [34]. The raw RNA-seq data of salt stress were obtained from the NCBI (PRJNA715099). The RNA-seq data of cold stress were obtained from our lab (unpublished, the raw data have been submitted to the BIG Data Center (http://bigd.big.ac.cn, (accessed on 18 September 2020)) with accession number CRA003229). The quality control of raw data and mapping to reference genomes were performed according to previous work [15]. In brief, the raw data were filtered using FastQC (http://www.bioinformatics.babraham.ac.uk/projects/fastqc/, (accessed on 2 July 2020)) to obtain clean reads. Clean reads were mapped to the reference genome of *Dendrobium catenatum* using the HISAT program [18,35]. The gene expression levels were calculated as FPKM (fragments per kilo bases of exons for per million mapped reads) using the software package StringTie [36]. The differential expression gene analyses with FDRs ≤ 0.05, log2 fold-change (FC) > 1 or log2 FC < −1, and with statistical significance (*p* value < 0.05) were using the edgeR package [37]. The differential expression genes were further subjected to a KEGG pathway enrichment analysis by KOBAS 2.0 [38].

### 4.4. Plant Materials, Drought Treatment, and Real-Time Quantitative PCR

Two-year-old cultivated *D*. *catenatum* were grown at 20 ± 2 °C with a light/dark cycle of 12/12 h and a 65–75% relative humidity as a control treatment. For drought treatment, the plants were not watered until the soil water content was approximately 10% under the condition of 25 ± 2 °C with a light/dark cycle of 12/12 h and a 40% relative humidity. After one day, the leaves were harvested with a four-hour interval in a day and immediately frozen in liquid nitrogen. 

Total RNA was isolated from leaf samples using a TransZol reagent (TransGen Biotech, Beijing, China). First-strand cDNA was reverse transcribed using the TIANscript RT Kit according to the manufacturer’s instructions (TransGen Biotech, Beijing, China). Quantitative real-time PCR analyses were performed using a SYBRGreen qPCR kit (TransGenBiotech) with a MyiQ system (Bio-Rad, Hercules, CA, USA), as described previously [12]. The primers for amplification are listed in Table S1.

## Figures and Tables

**Table 1:** The list of 8 *DcaNGLSs* in *D*. *catenatum*.

Gene Name	#ID	Length	MW(Da)	pI	Homology	#ID
*DcaDGD1*	Dca007046	794	90,572.2	7.78	AtDGD1	At3G11670
*DcaDGD2*	Dca012378	463	52,703.9	8.27	AtDGD2	At4G00550
*DcaMGD1*	Dca012806	455	50,087.8	9.07	AtMGD1	At4G31780
*DcaMGD2*	Dca023571	468	52,534.1	6.89	AtMGD2	At5G20410
*DcaMGD3*	Dca004841	504	55,877.5	5.91	AtMGD3	At2G11810
*DcaSQD1*	Dca007982	482	53,741.8	8.6	AtSQD1	At4G33030
*DcaSQD2*.1	Dca010090	303	34,626.8	7.66	AtSQD2	At5G01220
*DcaSQD2*.2	Dca010626	435	49,009.4	8.34	AtSQD2	At5G01220

## Data Availability

The data presented in this study are openly available in NCBI BioProject, accession number (PRJNA348403, PRJNA277909, PRJNA715099, PRJNA432825); BIG Data Center, accession number (CRA003229).

## Supplementary Materials

The following are available online at https://www.mdpi.com/article/10.3390/plants10061204/s1: Table S1: qRT-PCR primer list.
